# MicroRNA-133 Targets *Phosphodiesterase 1C* in *Drosophila* and Human Oral Cancer Cells to Regulate Epithelial-Mesenchymal Transition

**DOI:** 10.7150/jca.56138

**Published:** 2021-07-03

**Authors:** Ji Eun Jung, Joo Young Lee, Hae Ryoun Park, Ji Wan Kang, Yun Hak Kim, Ji Hye Lee

**Affiliations:** 1 Department of Life Science in Dentistry, School of Dentistry, Pusan National University, Yangsan 50612, Korea.; 2 BK21 FOUR Project, School of Dentistry, Pusan National University, Yangsan 50612, Korea.; 3 Dental and Life Science Institute, Pusan National University, Yangsan 50612, Korea.; 4 Department of Oral Pathology, School of Dentistry, Pusan National University, Yangsan 50612, Korea.; 5 Interdisciplinary Program of Genomic Science, Pusan National University, Yangsan 50612, Korea.; 6 Department of Anatomy, Department of Biomedical Informatics, School of Medicine, Pusan National University, Yangsan 50612, Korea.

**Keywords:** microRNA-133, *Drosophila melanogaster*, oral cancer, *PDE1C*, Epithelial-Mesenchymal Transition, mammal-to-*Drosophila*-to-mammal approach

## Abstract

Non-coding microRNAs (miRNAs) have been proposed to play diverse roles in cancer biology, including epithelial-mesenchymal transition (EMT) crucial for cancer progression. Previous comparative studies revealed distinct expression profiles of miRNAs relevant to tumorigenesis and progression of oral cancer. With putative targets of these miRNAs mostly validated *in vitro*, it remains unclear whether similar miRNA-target relationships exist *in vivo*. In this study, we employed a hybrid approach, utilizing both *Drosophila melanogaster* and human oral cancer cells, to validate projected miRNA-target relationships relevant to EMT. Notably, overexpression of *dme-miR-133* resulted in significant tissue growth in *Drosophila* larval wing discs. The RT-PCR analysis successfully validated a subset of its putative targets, including *Pde1c*. Subsequent experiments performed in oral cancer cells confirmed conserved targeting of human *PDE1C* by *hsa-miR-133*. Furthermore, the elevated level of miR-133 and its targeting of *PDE1C* was positively correlated with enhanced migrative ability of oral cancer cells treated with LPS, along with the molecular signature of a facilitated EMT process induced by LPS and TGF-β. The analysis on the RNAseq data also revealed a negative correlation between the expression level of *hsa-miR-133* and the survival of oral cancer patients. Taken together, our mammal-to-*Drosophila*-to-mammal approach successfully validates targeting of *PDE1C* by miR-133 both *in vivo* and *in vitro*, underlying the promoted EMT phenotypes and potentially influencing the prognosis of oral cancer patients. This hybrid approach will further aid to widen our scope in investigation of intractable human malignancies, including oral cancer.

## Introduction

Since its discovery in early 1990s, microRNA (miRNA) has been at the center of vibrant research activities in the field of human pathology 1. The function of miRNAs ranges from endogenous post-transcriptional regulators during development to oncomiRs involved in tumorigenesis and progression of various human malignancies, including oral cancer, via downregulation of tumor suppressors. Despite continuous efforts in research, the 5-year survival rate of patients suffering from oral cancer, mostly oral squamous cell carcinoma (OSCC), remains relatively low around 50% compared to other malignancies 2, 3. With its incidence rate among the population younger than 45 years fast rising worldwide 4, it requires development of novel therapeutic options with improved efficacy. In an attempt to achieve this goal, a growing body of research efforts has been made to unravel pathologic modulation of miRNAs and their targets that may underlie tumorigenesis and progression of oral cancer. Indeed, recent findings address abnormal regulation of miRNAs in disease states from case-control studies or in cancer cell lines *in vitro* 5, 6. With the computational algorithms implemented in the publicly available database, putative targets of a single miRNA can be predicted based upon identification of miRNA-binding sites within the 3'-UTR region of its targets 7-9. Following prediction, subsequent validation steps of miRNA-target relationships have been mostly performed *in vitro*. Thus, whether similar miRNA-dependent targeting occurs *in vivo* remains largely undetermined. It should also be noted that commonly used target prediction platforms could yield many false positives 10.

Among candidate miRNAs with potential diagnostic and prognostic value for oral cancer, we focus on miR-133 in this study. miR-133 has been one of the most extensively investigated miRNAs for its roles in development of skeletal and heart muscle diseases as well as various types of cancer 11. miR-133 is known to participate in embryonic myogenesis via targeting *Gli3*, a transcriptional repressor of the Hedgehog pathway 12 as well as in osteogenesis via targeting *Runx2*, a bone morphogenetic protein (BMP) response gene crucial for bone formation 13. Further molecular targets of miR-133 include, but not limited to, epidermal growth factor receptor (*EGFR*) 14, fibroblast growth factor 1 (*FGF1*) 15 and insulin-like growth factor-1 receptor (*IGF1R*) 16. Regulation of these targets and its functional consequences in tumorigenesis have suggested miR-133 as a potential tumor suppressor in gastric cancer 17, glioma 18 and non-small cell lung cancer 19.

Recent studies indicate a role of miR-133 in cancer progression by regulating the cellular process named epithelial-mesenchymal transition (EMT), a critical phenomenon underlying invasion and migration of cancer cells 20. Regulation of EMT process by miR-133 was first demonstrated in cardiac myocytes 21. In their study, Muraoka et al. demonstrated miR-133-dependent cardiac reprogramming by its targeting of *Sai1*, a key regulator of EMT 21. Later studies have indicated similar miR-133-dependent regulation of EMT implicated in human cancer. For instance, downregulation of *EGFR*, *Forkhead box C1* (*FOXC1*) and *Forkhead box Q1* (*FOXQ1*) by miR-133 was thought to mediate reduced cell proliferation, migration and invasion of prostate cancer 14, pituitary adenoma 22 and lung cancer 23, respectively. miR-133-dependent targeting of *Presenilin 1* (*PSEN1*) was also linked to the diminished ability of gastric cancer cells to undergo EMT 24. Meanwhile, there have been only a few studies focused on miR-133-dependent suppression of cancer progression in esophageal squamous cell carcinoma and OSCC, which identified *COL1A1* as a presumed target to control invasive and migratory behaviors of cancer cells 25, 26. As demonstrated in these studies, however, validation platforms for miR-133 targets in regard to oral cancer and its progression remain mostly restricted to *in vitro* settings, thus prompting us to unravel additional targets regulated by miR-133 *in vivo*.

In this study, we introduce an alternative animal model, *Drosophila melanogaster*, in order to decipher functional relationships between miRNA, specifically miR-133, and its unverified targets *in vivo*. With high homology to human disease-related genes and relevant signaling pathways of less redundancy 27, 28, *Drosophila* poses itself as an attractive animal model equipped with sophisticated genetic approaches. We took advantages of this model organism to investigate the relationship between miR-133 and its putative targets *in vivo*, by forcing its expression in *Drosophila* larval tissues and by monitoring the transcript level of its putative targets. In order to intensify the clinical significance of our findings in *Drosophila*, we adopted a hybrid approach to conduct parallel experiments using oral cancer cells in culture. Our findings indicate a shared targeting of *phosphodiesterase 1C* (*PDE1C*), a member of phosphodiesterase family, by miR-133 in both *Drosophila* tissues and human oral cancer cells, underlying promoted tissue growth and more pronounced development of EMT phenotypes. Taken together, our study presents another example of successful application of a mammal-to-*Drosophila*-to-mammal paradigm that allows us to functionally validate human miRNA-dependent regulation of their putative targets. Such combinatorial approach will further help to broaden our understanding of the pathophysiology of oral cancer.

## Materials and methods

### FIy stocks

All crosses and stocks were kept on a standard medium at 24°C with humidity between 40% and 60%. The fly stocks used in our study were acquired from the Bloomington Drosophila Stock Center, including *w^1118^; P{UAS-LUC-mir-133.T} attP2*, w^*^; M{UAS-mir-133.Sb} ZH-51D/CyO and w^*^; M{UAS-mir-133.Sb} ZH-86Fb/TM3, Sb Ser, and the Vienna Drosophila Resource Center for *UAS-Pde1c-shRNA* (#101906). The 459.2-GAL4 driver (w^*^; P{GawB}459.2) was used for expression of *UAS-miRNA-133* transgenes specifically in *Drosophila* imaginal wing discs.

### lmage analysis of *Drosophila* imaginal wing discs

Imaginal wing discs dissected from the wondering third instar larvae in HL3.1 saline were fixed in PBS containing 3.7% formaldehyde for 20 minutes and mounted in Vectashield^®^ Antifade Mounting Media (Vector Laboratories, Burlingame, CA, USA). The images were captured with an Olympus microscope (EX51, Olympus, Center Valley, PA, USA) and further processed with Adobe^®^ Photoshop CS6 (Adobe Corporation, San Jose, CA, USA). The ImageJ package (National Institutes of Health (NIH), Bethesda, MD, USA) was used for measurements of overall size of individual wing discs.

For confocal microscopy, individual discs following fixation were permeabilized with PBS containing Triton X‐100 and incubated with Alexa Fluor® 555 Phalloidin (at 1:20, Cell Signaling Technology) at room temperature for 1 hour before their mounting onto a slide for scanning. The images were taken with a confocal microscope (LSM700, Carl Zeiss, Jena, Germany) and processed with Zen (Carl Zeiss), Image J (NIH) and Adobe^®^ Photoshop CS6 (Adobe Corporation).

### Cell culture

All cells were maintained at 37°C in a humidified incubator supplied with 5% CO_2_. The human oral squamous cell carcinoma (OSCC) cell lines, including SAS and OSC20, were cultured in Dulbecco's Modified Eagle's Medium and Ham's nutrient mixture F12 (Hyclone, Logan, UT, USA) supplemented with 10% fetal bovine serum (10% FBS; GIBCO, ThermoFisher Scientific, Waltham, MA, USA) and penicillin-streptomycin (100 units/ml, Invitrogen, ThermoFisher Scientific). These cells were treated with Plasmocin™ (InvivoGen, San Diego, CA, USA) for three weeks to remove potential mycoplasma contamination. For stable cell lines expressing miR-133, the pMSCV-puro retroviral vector (Takara Bio Inc, Kusatsu, Japan) was used for subcloning of miR-133. The Phoenix™ packaging cells (ThermoFisher Scientific) were then transfected with 10 μg of the prepared retroviral vector. After being infected with retrovirus, OSCC cells expressing the ectopic miR-133 were selected via an application of puromycin (2 μg/ml, Invitrogen). Stable cell lines were then further treated with Plasmocin™ (InvivoGen) for three more weeks. Lipopolysaccharides (LPS) derived from a strain of *Escherichia coli* (*E. coli* O111:B4; Sigma-Aldrich Inc., St. Louis, MO, USA) was dissolved in DPBS and prepared to a concentration of 5 μg/μl. Transforming growth factor (TGF)-β1 (R&D Systems, Minneapolis, MN, USA) was dissolved to prepare a stock solution (20 μg/mL) and used at a concentration of 5 μg/μl. 3-Isobutyl-1-methylxanthine (IBMX), a non-selective cAMP/cGMP phosphodiesterase inhibitor, was dissolved and used at a concentration of 1 mM. OSCC cell lines initially grown with 10% FBS were exposed to 0.5% FBS for 4 hours before 48 hour-long treatments of LPS, TGF-β1 or IBMX.

### Reverse Transcription Polymerase Chain Reaction (RT-PCR)

Total RNA was extracted from a total of 5~10 wing discs of the third instar *Drosophila* larvae using the TRIzol reagent (Invitrogen), followed by a step of cDNA synthesis using TOPscript™ cDNA synthesis kit (Enzynomics, Inc., Daejeon, Korea). A standard RT-PCR reaction was performed using PrimeScript RT Master Mix (Takara Bio Inc.), according to the manufacturer's instruction. Each RT-PCR reaction was analyzed with gel electrophoresis and visualization using Multiple Gel DOC system (Fuji Photo Film Co., Ltd., Tokyo, Japan).

### Real-time quantitative RT-PCR analysis

The real-time quantitative RT-PCR reaction was performed using TOPreal™ SYBR Green qPCR master mix (Enzynomics, Inc.). The reaction cycles consisted of the following steps: 1 cycle of initial denaturation (95°C for 10 minutes) and 40 cycles of denaturation (95°C for 15 seconds), annealing (60°C for 30 seconds) and extension steps (72°C for 30 seconds). The quantitative measurement of each transcript was carried out using 7500 Real-Time PCR Instrument System (Applied Biosystems, ThermoFisher Scientific). The mRNA levels of *Drosophila actin5c* and human *36B4* served as internal controls for normalization in *Drosophila* and human oral cancer cell experiments, respectively. Each reaction was completed in triplicates. The primer sets used include: *Drosophila Pde1c*, ATCTTTGTGGACCGCATGTATC as a forward primer and GAACTTGTGAATCGAGCCGTA as a reverse primer; *Drosophila actin5c*, CCACACCGTCCCCATCTATG as a forward primer and AGTCCAGGGCAACATAGCAC as a reverse primer; human *PDE1C-1*, TCTCAAAGGATGACTGGAGG as a forward primer and GCTTCTCTGTCACCCTGTC as a reverse primer; human *PDE1C-2*, TTCGGCAACATCTCATGGCT as a forward primer and GGAATGGCTGAATTGGCGTC as a reverse primer; human *E-cadherin*, CATCTTTGTGCCTCCTGAAA as a forward primer and TGGGCAGTGTAGGATGTGAT as a reverse primer; human *N-cadherin*, CCTGCTTATCCTTGTGCTGA as a forward primer and CCTGGTCTTCTTCTCCTCCA as a reverse primer; human *36B4*, CACCATCTTCCAGGAGCGAG as a forward primer and GACTCCACGACGTACTCAGC as a reverse primer.

For quantitative analyses of miR-133 in SAS cells with stable expression of miR-133, total RNA isolated from 6-well plates was converted into cDNA by TaqMan^®^ MicroRNA Reverse Transcription Kit (Applied Biosystems) and quantified by TaqMan^®^ MicroRNA Expression Assays (Applied Biosystems) according to the manufacturer's instructions. Once normalized to the level of *RNU48*, the relative expression level of miR-133 was determined as fold changes. All TaqMan^®^ MGB probes were purchased from Applied Biosystems (*RNU48* and *hsa-miR-133a*, #442797S).

### Transfection of *PDE1C* siRNA

For knockdown of *PDE1C* in OSCC cells, we designed short-interfering RNAs (siRNAs) with the following sequence: 5137-1 (Human, *PDE1C*), CUGUCAUCUCCGUUGACUA and UAGUCAACGGAGAUGACAG; 5137-2 (Human, *PDE1C*), CACAUCAAUCGGGAGAGAU and AUCUCUCCCGAUUGAUGUG; 5137-3 (Human, *PDE1C*), GACUAGAGCAUACCGGAA and UUCCGGUAUGCUCGUAGUC (Bioneer Co, Ltd, Daejeon, South Korea). The corresponding scrambled siRNA was also purchased from the same source (Bioneer) and used as controls. Transfection of siRNAs was performed using XtremeGENE™ siRNA Transfection Reagent (Sigma-Aldrich Inc.), at two different concentrations (low and high concentrations of siRNA with 20 ng and 50 ng of total amount, respectively).

### Wound-healing assay

The migratory ability of oral cancer cells was assessed with a scratch wound assay. Briefly, SAS OSCC cells were cultured in six-well plates (2×10^5^ cells per well) to 90% confluence, serum-starved for 4 hours and gently scrapped with a P-200 pipette tip to produce a clean wound area at the center of the cell monolayer. These cells were then treated with LPS at a concentration of 5 μg/μl for 24 hours. The wound closure areas were visualized at 0 and 24 hours of incubation under a phase-contrast microscope with a magnification of 100.

### Western blot analysis

Cells were lysed in RIPA buffer (Elpis-Biotech, Inc., Daejeon, Korea), supplemented with phosphatase inhibitor and protease inhibitor cocktails (Sigma-Aldrich Inc.). The amount of protein in the lysate was measured using Pierce BCA protein assay (ThermoFisher Scientific). Proteins from each lysate were separated on an SDS-PAGE gel and transferred onto nitrocellulose membranes (MilliporeSigma, Burlington, MA, USA), followed by a blocking step with 3% BSA. The primary antibodies used include: β-Actin (Santa Cruz Biotechnology, Dallas, TX, USA), vimentin (D21H3), Slug (c19G7), Snail (C15D3), N-cadherin (D4R1H), E-cadherin (24E10), ZO-1 and claudin-1 (D5H1D) (Cell Signaling Technology, Danvers, MA, USA). All primary antibodies were used at a concentration of 1:1000. The secondary antibodies for chemiluminescence were obtained from Cell Signaling Technology. Chemiluminescence was visualized with WesternBright™ ECL (Advansta Inc., San Jose, CA, USA) and WesternBright™ Sirius kit (Advansta Inc.).

### Immunofluorescent staining and imaging of OSCC cells

SAS OSCC cells were plated at a density of 2×10^4^ cells per well in an 8‐well Lab‐Tek II Chambered Coverglas (SPL Life Sciences, Pocheon-si, Korea), incubated for 24 hours and treated with TGF-β1 for up to 48 hours. Following incubation, these cells were fixed in PBS containing 4% paraformaldehyde at room temperature for 15 minutes. Fixed cells were then washed twice, permeabilized with PBS containing Triton X‐100 and blocked with 1% BSA for 1 hour on a locking shaker. The primary antibodies against E-cadherin and claudin-1 (Cell Signaling Technology) were used for imaging at a concentration of 1:100. SAS cells were then incubated with the fluorescein isothiocyanate‐conjugated secondary antibody and mounted in Vectashield^®^ Antifade Mounting Medium with DAPI (Vector Laboratories). The images were taken with a confocal microscope (LSM700, Carl Zeiss) and processed with Image J (NIH) and Adobe^®^ Photoshop CS6 (Adobe Corporation).

### Gene expression and survival analysis of TCGA collection

The RSEM normalized RNAseq data and RPKM normalized miRNA data of OSCC were selected from Broad GDAC Firehose (https://gdac.broadinstitute.org/) for subdivisions of TCGA-HNSC according to the following anatomic sites of primary tumors: lip, tongue, floor of mouth, buccal mucosa, hard palate, oropharynx, tonsil, and oral cavity (unspecified). The analyzed dataset consisted of 236 control and 145 OSCC tumor samples. The expression levels of human miR-133s (miR-133a-1 (miR-133a-5p), miR-133a-2 (miR-133a-3p) and miR-133b) and PDE1C in the control and OSCC samples were compared using a Mann-Whitney-Wilcoxon test with Bonferroni correction based on the statannot python package (statannot version 0.2.2 and python version 3.7.1, Python Software Foundation, 2020). Subsequently, the survival analyses were performed using the lifelines python package (lifelines version 0.24.0, Python Software Foundation, 2020).

### Statistical analysis

All quantitative RT-PCR experiments each consisting of triplicates were performed at least three times for statistical analyses. The data from two independent groups were compared and analyzed with unpaired two-tailed Student's *t*-test. For multiple comparisons among three groups or more, one-way analysis of variance (ANOVA) was conducted using OriginPro 2020b (OriginLab Corporation, Northampton, MA, USA), followed by Tukey's post-hoc tests. For quantification of western blot analyses, one-way ANOVA, non-parametric Kruskal-Wallis ANOVA followed by Dunn's test (both for multiple comparisons among three groups or more), or Mann-Whitney-Wilcoxon test (for two groups) was performed using OriginPro 2020b (OriginLab Corporation). For analyses of the RNAseq datasets, a Mann-Whitney-Wilcoxon test was conducted with Bonferroni correction using the python packages (Python Software Foundation, 2020). The *P* value less than 0.05 were considered statistically significant.

## Results

### Overexpression of miR-133 promoted tissue growth in *Drosophila* wing discs

Recent studies have documented differential expression profiles of individual microRNAs (miRNAs) between normal vs. oral cancer tissues as well as oral cancer cell lines, yielding a list of candidates potentially linked to oral cancer, including miR-21, miR-34, miR-133 and miR-203 29-32. Among these miRNAs, miR-133 has been mostly proposed as a tumor suppressor implicated in various human malignancies, including oral cancer 17-19, 33, 34. However, a causative relationship between reduced expression of miR-133 and tumorigenesis or progression of oral cancer has not been well established. In this study, we took advantage of the *Drosophila* system that would enable monitoring of functional consequence of genetic manipulations *in vivo* with ease, in addition to the existence of homologous miRNAs with the shared seed sequence. In order to investigate the role of miR-133 in tissue growth, we induced overexpression of miR-133 in *Drosophila* tissues. The larval imaginal wing discs were selected for this purpose, as they are representative of an epithelial origin, suitable for studying molecular mechanisms underlying the pathophysiology of oral squamous cell carcinoma (OSCC), the most common type of oral cancer. The wing discs have been proven as a useful platform to study a number of cellular signaling pathways important for tumorigenesis and cancer progression, including those involved in the planar cell polarity and the Hedgehog signaling 35, 36.

**Figure 1:**
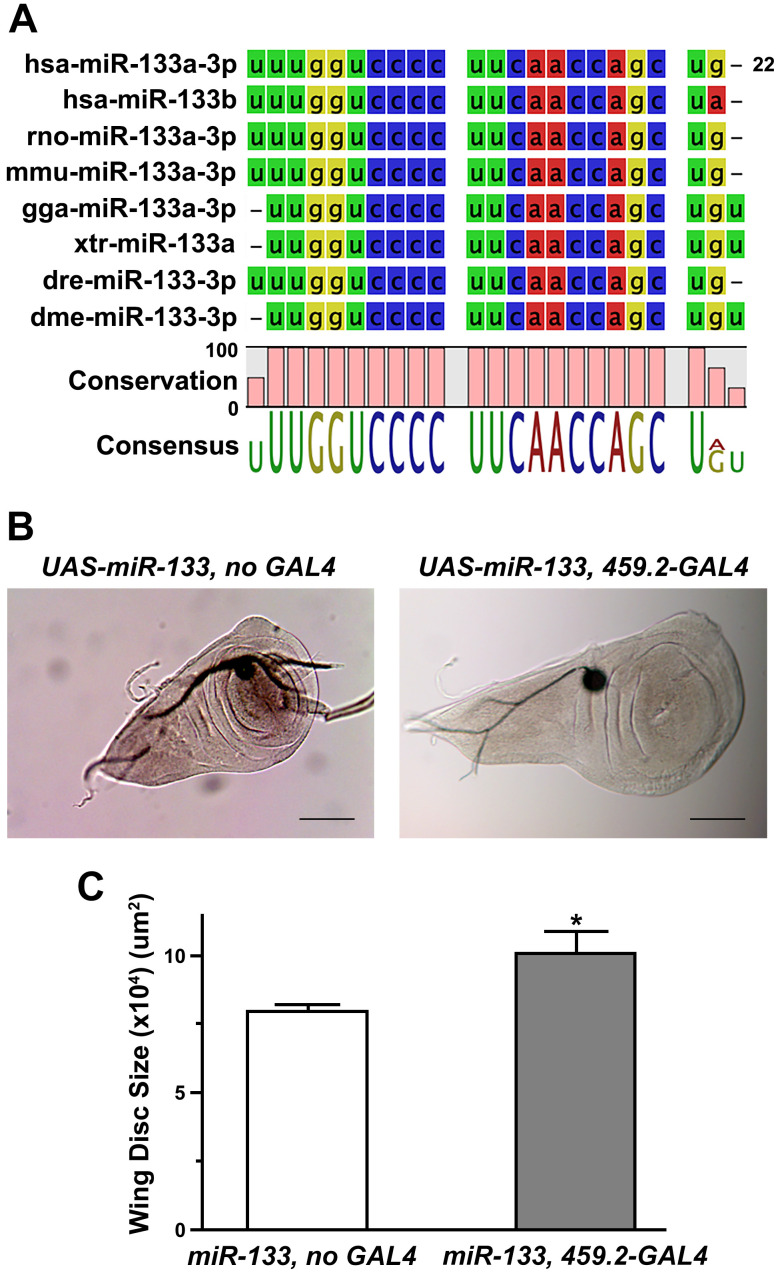
** Promoted tissue growth induced by overexpression of *Drosophila* miR-133.** (A) The sequences of mature miRNAs are compared between *Drosophila* (*dme-miR-133*) and various mammalian miR-133s (*hsa, Homo sapiens*;* rno, Rattus norvegicus*;* mmu, Mus musculus*;* gga, Gallus gallus*;* xtr, Xenopus tropicalis*;* dre, Danio rerio*). Conserved sequences are indicated at the bottom of each comparison. (B) Representative images of *Drosophila* wing discs are shown for those with (right) and without overexpression of miR-133 (left). Scale bar, 100 μm. (C) The pooled data are shown for measurements of the size of wing discs in each genotype indicated. The number of wing discs examined: 9 and 18 for *UAS-miR-133* with and without *459.2-GAL4*, respectively. Mean ± SEM values are indicated. *, *P*<0.05.

When the sequence of mature *Drosophila* miR-133 (*dme-miR-133-3p*) was compared with those of multiple vertebrate miR-133s, including human miR-133s, we found a high degree of conservation (Figure 1A), further confirming the validity of the *Drosophila* system. Importantly, overexpression of miR-133 in *Drosophila* wing discs using the GAL4-UAS system 37 resulted in a significant increase in their size, compared to the controls (Figures 1B and 1C), indicating miR-133 as a factor favoring tissue growth. To examine the nature of promoted growth of the wing discs, we monitored the size of cells constituting the discs. Our results indicated no significant change in the cell size (Supplementary Figure S1), suggesting enhanced cell proliferation corresponding to the increased size of wing discs. It is important to note that our results of miR-133-induced promotion of tissue growth are inconsistent with a proposed role of miR-133 as a tumor suppressor based upon previous clinical and *in vitro* experimental studies.

### Overexpression of miR-133 reduced the transcript level of its predicted target, *PDE1C*, in both *Drosophila* wing discs and human oral cancer cells

**Figure 2:**
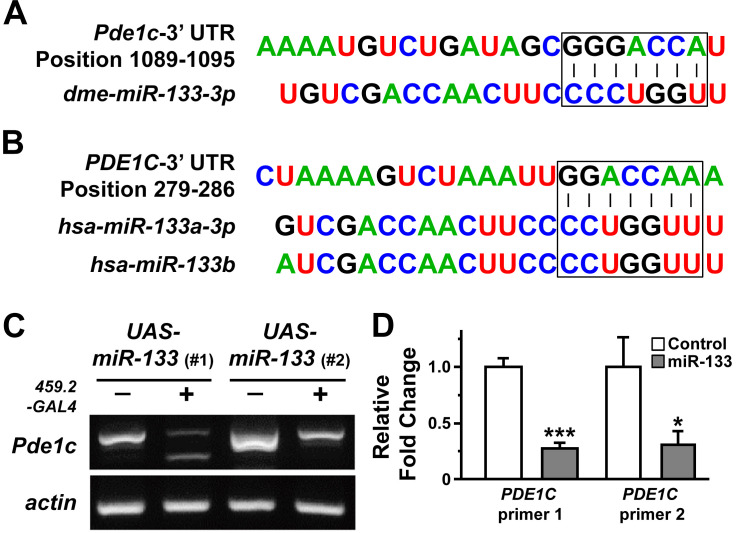
** Downregulation of *PDE1C* by overexpression of miR-133 in both *Drosophila* wing discs and human oral cancer cells.** (A and B) The putative target sites of miR-133 are shown within the 3'-UTR regions of *Drosophila* (A) and human *PDE1C* (B). (C) A representative result of RT-PCR reactions is shown to visualize miR-133-dependent downregulation of miR-133 in *Drosophila* wing discs. Two independent lines of *UAS-miR-133* (#1 and #2) are used for comparison. (D) The pooled data from quantitative RT-PCR analyses are shown for visualization of miR-133-dependent downregulation of *PDE1C* in a human oral cancer cell line, SAS cells, with stable expression of miR-133. Two independent sets of primers specific for *PDE1C* are used for analysis. Mean ± SEM values are indicated for experiments repeated three times. Two-sample *t*-test is performed. *, *P*<0.05 and ***, *P*<0.001 for control vs. miR-133.

Our results on tissue growth following overexpression of miR-133 prompted us to investigate putative targets of miR-133 that might mediate promoted tissue growth in *Drosophila* wing discs. The putative targets of miR-133 predicted by computational algorithms include an array of molecules ranging from those involved in embryonic myogenesis to key players in cancer biology. For functional validation of miRNA-target relationship *in vivo*, we first attempted to identify putative targets of miR-133 from TargetScanFly database (release 6.2 and 7.2 in 2012 and 2018, respectively). Upon isolation of putative targets, RT-PCR analyses were performed in larval wing discs overexpressing miR-133 with individual primers specific for each target. Among the candidates screened for changes in their mRNA level, we found a prominent reduction in the amount of *Pde1c* transcript, with a conserved target site identified within its 3'-UTR region (Figures 2A and 2C). In line with miR-133-dependent downregulation of *Pde1c* at the level of mRNA, an implementation of RNA interference against *Pde1c* resulted in a modest growth of the wing discs, albeit a lack of statistical significance (Supplementary Figure S2), suggesting a partial contribution of miR-133-dependent targeting of *Pde1c* to promoted tissue growth.

With relatively limited similarity at the level of *PDE1C* mRNA (67% identity with 17% of gene coverage) and its protein product (58% identity with 53% coverage), we investigated the possibility of a conserved targeting of *PDE1C* transcript by human miR-133 in oral cancer cells, including SAS and OSC20 OSCC cell lines. Importantly, *PDE1C* was also listed as a putative target of human miR-133 (*hsa-miR-133a-3p* and *hsa-miR-133b*) according to TargetScan Human database (release 6.2 and 7.2 in 2012 and 2018, respectively). The prediction algorithms indeed identified a potential miR-133-binding site within the 3'-UTR region of human *PDE1C* (Figure 2B). As the first step of visualizing miR-133-dependent regulation of *PDE1C*, we monitored the level of endogenous miR-133 in oral cancer cells. Our results indicated extremely low level of miR-133 in OSCC cells, reaching nearly a detection limit of a quantitative RT-PCR analysis (Ct value ranging from 35 to 37). Based upon this finding, we sought to establish cell lines with stable expression of miR-133, thus providing us a better resolution to dissect potential miR-133-dependent regulations of cellular responses in subsequent experiments. When the level of miR-133 was increased nearly 30-fold (Supplementary Figure S3), we found a significant reduction in the level of *PDE1C* transcripts in these OSCC cells, as confirmed by two independent sets of primers specific for *PDE1C* (Figure 2D). Together with our findings in *Drosophila*, these results thus demonstrate a shared targeting of *PDE1C* by miR-133 in both *in vivo* and *in vitro* across different phyla.

### Overexpression of miR-133 displayed molecular and behavioral signatures representing facilitated epithelial-mesenchymal transition in oral cancer cells

**Figure 3:**
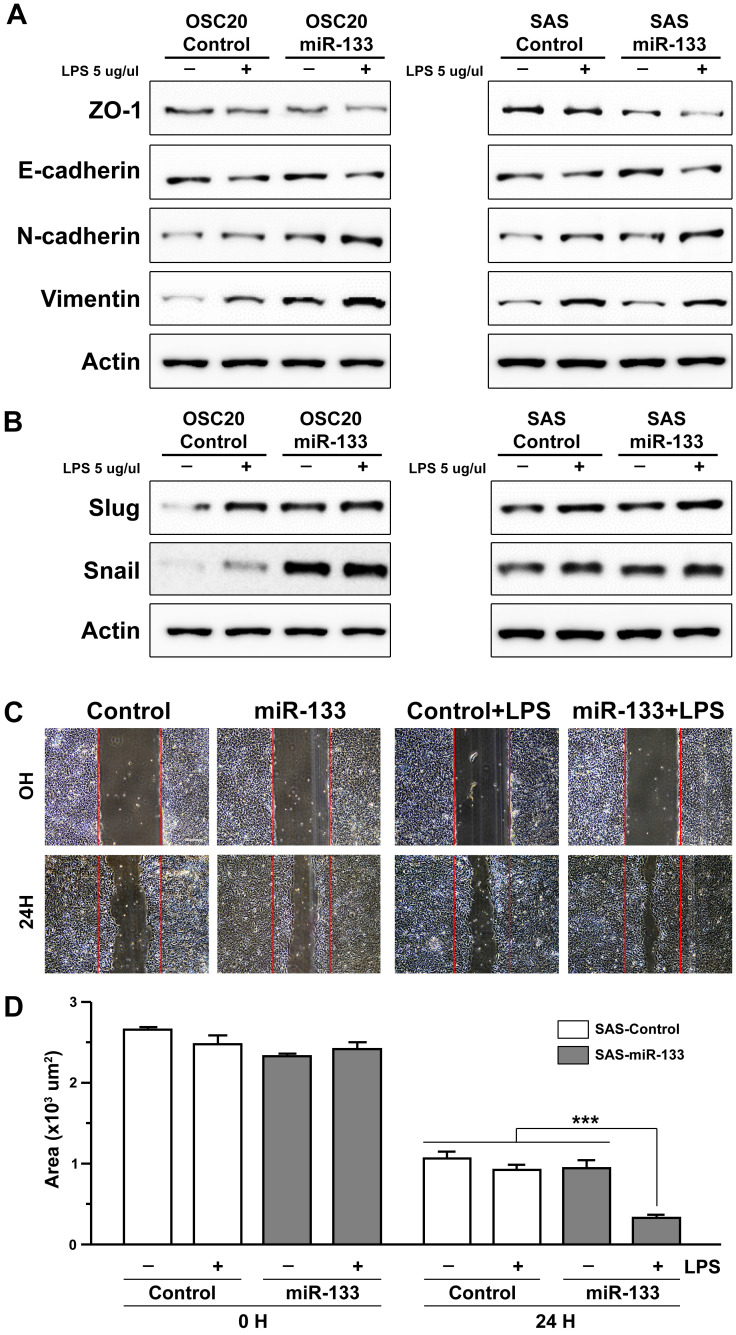
** miR-133-dependent facilitation of LPS-induced epithelial-mesenchymal transition.** (A and B) The levels of molecular markers characteristic of EMT are indicated by western blot analyses upon an exposure of two independent oral cancer cells, OSC20 (left) and SAS (right) to LPS, in the presence (miR-133) and absence of stable expression of miR-133 (control). (C) The artificially generated gap regions of cultured SAS cell clusters with (miR-133) and without stable expression of miR-133 (control) are shown before (0H) and after 24 hour-long treatments of LPS (24H). The initial gap generated by scrapping is indicated with red lines in each image. Scale bar, 10 µm. (D) A quantitative measurement of the wound area is shown for each group before and after a 24 hour-long treatment of LPS. Mean ±SEM values are indicated for six different regions in cultures. One-way ANOVA test is performed. ***, *P*<0.001 for comparisons among the experimental conditions indicated.

The involvement of miR-133 in the process of cancer progression has been documented in previous studies on esophageal and oral cancer 25, 26, mostly suggesting its role as a factor inhibiting a cellular process named epithelial-mesenchymal transition (EMT) 20. In the present study, we investigated the effect of miR-133 on molecular networks functioning in the process of EMT in oral cancer cells. We first monitored the effect of miR-133 on proliferation of SAS OSCC cells with its stable expression and found no evidence of promoted cell proliferation (Supplementary Figure S4). Next, two independent OSCC cell lines, OSC20 and SAS cells, were treated with lipopolysaccharide (LPS) derived from *E. coli* in order to facilitate visualization of miR-133-dependent regulation of EMT. A 48 hour-long treatment of LPS was capable of expressing a molecular profile characteristic of EMT 38 (Figure 3). For instance, representative cell surface molecules such as ZO-1 were significantly downregulated, with a similar trend found in E-cadherin as well. In contrast, the expression of mesenchymal cell-biased markers such as N-cadherin and vimentin was significantly promoted in oral cancer cells (Figure 3A). Furthermore, Slug and Snail, two important transcriptional regulators of EMT 39, were significantly upregulated upon an exposure to LPS (Figure 3B), indicating a successful induction of EMT by LPS in oral cancer cells. Importantly, stable expression of miR-133 in these cells appeared to further accelerate phenotype of EMT, both in the presence and absence of LPS, with subtle differences between two oral cancer cell lines tested (Figures 3A and 3B). The quantitative measurement of the protein level from multiple western blot analyses also exhibited similar trends in these EMT probes, albeit with limited statistical significance (Supplementary Figures S5 and S6). These results thus suggest a facilitated EMT process by enhanced expression of miR-133 in oral cancer cells.

The positive correlation between the activation of EMT process and acquisition of invasive ability of cancer cells has been well documented in previous studies (see Son and Moon, 2010, for a review) 40. With an enhanced EMT phenotype in oral cancer cells upon stable expression of miR-133 (Figures 3A and 3B), we sought to investigate whether such molecular signature would coincide with changes in cellular behaviors. For this purpose, a migratory potential of oral cancer cells was examined with a wound healing assay, through which the width of an artificially generated gap within the cluster of cultured cells was monitored for a specified duration of time. While a treatment of OSCC cells with LPS alone did not resulted in a significantly faster closure of the gap, the remaining wound area became significantly narrower in LPS-treated OSCC cells with stable expression of miR-133 (Figures 3C and 3D for quantification), in line with more prominent molecular characteristics of EMT (Figures 3A and 3B; Supplementary Figures S5 and S6). Such behavioral change in cellular migratory potential further confirms a facilitated EMT process and indicates acquisition of more aggressive potential in oral cancer cells with upregulated miR-133.

**Figure 4:**
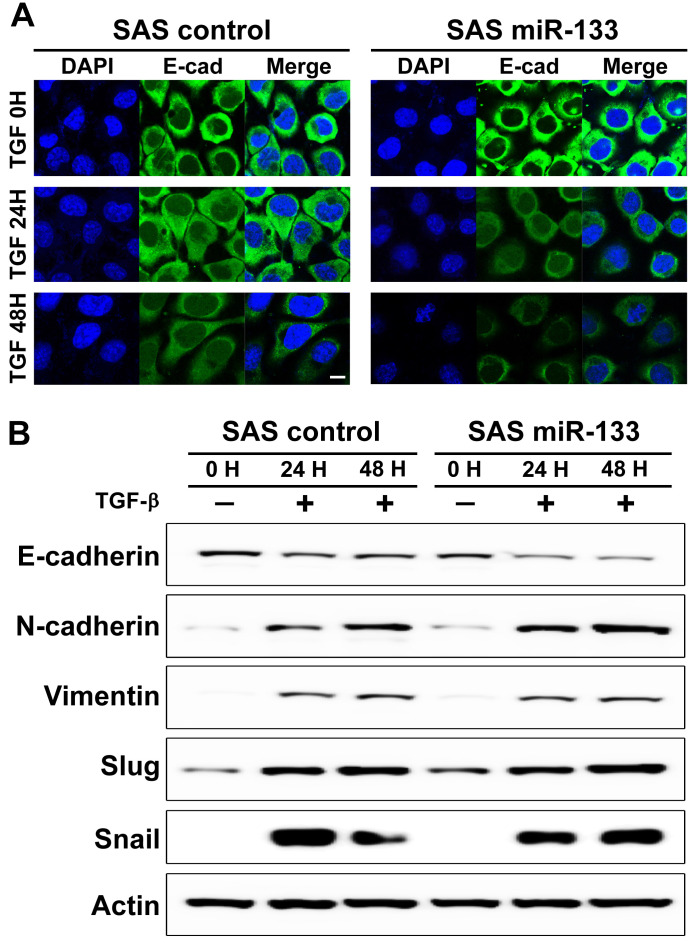
** miR-133-dependent facilitation of TGF-β-induced epithelial-mesenchymal transition in oral cancer cells.** (A) Representative images of confocal microscopy are shown for visualization of E-cadherin following an exposure of SAS oral cancer cells to TGF-β for up to 48 hours, in the presence (miR-133) and absence of stable expression of miR-133 (control). Scale bar, 10 µm. (B) The levels of molecular markers characteristic of EMT are indicated by a western blot analysis upon an exposure of SAS cells to TGF-β, in the presence (miR-133) and absence of stable expression of miR-133 (control).

In addition to LPS, an application of transforming growth factor (TGF)-β to cancer cells has been mapped to promoted EMT phenotypes in a variety of human malignancies 41, including oral cancer 42. As expected, we found a prominent downregulation of E-cadherin, an epithelial cell-biased marker, from the boundary of oral cancer cells treated with TGF-β (Figure 4A, left panels). A similar trend of downregulation was also evident in the distribution of claudin-1, another well-established epithelial marker (Supplementary Figure S7). Further analysis of a western blot assay confirmed the molecular signature representative of EMT (Figure 4B). Notably, OSCC cells stably expressing miR-133 displayed a more prominent decrease in the immunoreactivity against E-cadherin compared to the controls (Figure 4A, right vs. left panels), suggestive of a facilitated EMT process. Enhanced EMT phenotypes were confirmed with a subsequent western blot analysis, demonstrating significantly reduced expression of E-cadherin in combination with a trend of elevated levels of vimentin, Slug and Snail (Figure 4B; Supplementary Figure S8 for quantitative analyses). It should be noted that there was no evidence of promoted cell proliferation in TGF-β-treated SAS OSCC cells with stable expression of miR-133, in comparison with the control cells (Supplementary Figure S4), suggesting a minimal to no influence of miR-133 upon proliferative potentials of these cells. Together with our findings in LPS-treated OSCC cells, these results thus imply miR-133 as an important regulator of cancer progression by facilitating the EMT process.

### Downregulation of *PDE1C* was positively correlated with the molecular signature of facilitated EMT in oral cancer cells

**Figure 5:**
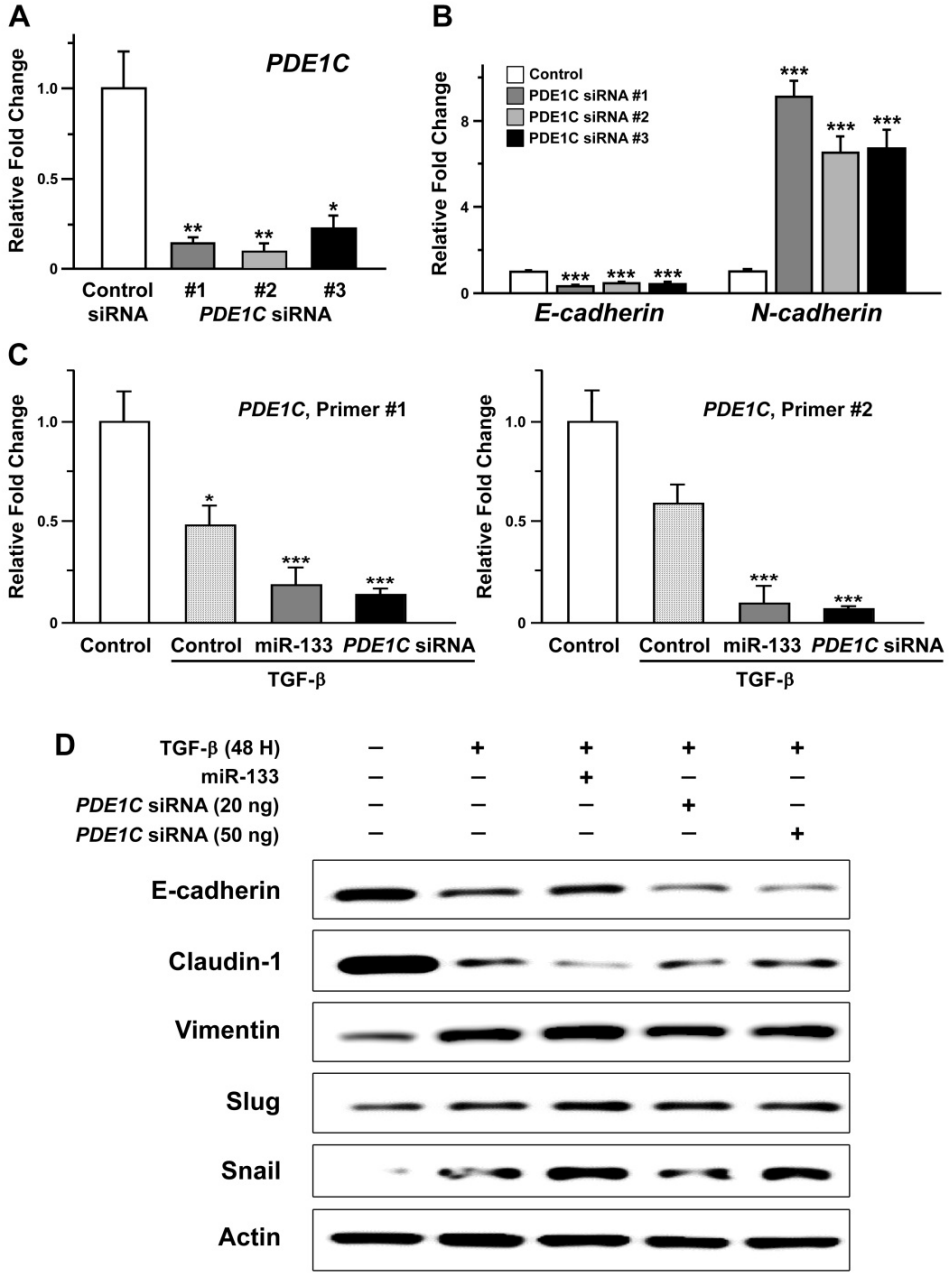
** Facilitated epithelial-mesenchymal transition induced by downregulation of PDE1C in oral cancer cells.** (A) The relative levels of *PDE1C* mRNAs are shown for SAS cells with transfection of three independent short-interfering RNAs (siRNAs) against *PDE1C*. Mean ± SEM values are indicated for experiments repeated three times. One-way ANOVA test is performed. *, *P*<0.05 and **, *P*<0.01 for control siRNA vs. other groups. (B) The transcript levels of *E-cadherin* and *N-cadherin* are compared in SAS cells following transfection of three independent siRNAs against *PDE1C*. Mean ± SEM values are indicated for experiments repeated three times. One-way ANOVA test is performed. ***, *P*<0.001 for control siRNA vs. other groups. (C) The transcript levels of *PDE1C* are measured upon an exposure of SAS cells to TGF- β, in the presence (miR-133) and absence of stable expression of miR-133 (control) as well as with transfection of *PDE1C* siRNAs. Two independent sets of primers specific for *PDE1C* are used for analysis. Mean ± SEM values are indicated for experiments repeated three times. One-way ANOVA test is performed. *, *P*<0.05 and ***, *P*<0.001 for control vs. other groups. (D) The levels of molecular markers characteristic of EMT are indicated by a western blot analysis upon an exposure of SAS cells to TGF-β, in the presence (miR-133) and absence of stable expression of miR-133 (control) as well as with transfection of *PDE1C* siRNAs at two different concentrations.

A previous study has suggested a potential implication of PDE1C in regulation of the EMT process in glioblastoma multiform cells 43. Our results indicating miR-133-dependent downregulation of PDE1C and facilitated EMT phenotypes prompted us to hypothesize the idea that the level of PDE1C would be correlated with development of EMT phenotypes. To test this hypothesis, we designed short-interfering oligonucleotides (siRNAs) against *PDE1C* for its downregulation in OSCC cells. Transfection of three independent *PDE1C*-siRNAs resulted in effective reduction of its transcript levels (Figure 5A). Interestingly, a subsequent quantitative RT-PCR analysis revealed siRNA-induced changes in the level of *E-cadherin* and *N-cadherin* mRNAs (Figure 5B). However, we failed to detect changes at the level of their protein products as well as other EMT markers when visualized with western blot analyses (Supplementary Figure S9), suggesting a relatively limited effect of *PDE1C*-siRNA itself on regulation of EMT phenotype in the absence of triggering events.

We then investigated whether the level of PDE1C could be positively correlated with development of EMT triggered by a stimulus such as TGF-β in OSCC cells (Figure 4). Notably, an application of TGF-β to OSCC cells induced a prominent decrease in the level of *PDE1C* mRNAs, as shown with two independent primers specific for *PDE1C* (Figure 5C, dotted pattern). A similar treatment of TGF-β in OSCC cells stably expressing miR-133 displayed a further decrease in the level of *PDE1C* transcripts (Figure 5C, dark gray), consistent with our data demonstrating a more facilitated EMT phenotype under the same condition (Figure 4B). These data allowed us to postulate a hypothesis that PDE1C might play an important role in mediating the EMT signaling.

Such idea was further supported by our finding demonstrating *PDE1C* siRNA-induced changes in molecular markers characteristic of EMT. In details, a total of 20 or 50 ng of *PDE1C* siRNAs, containing a mixture of two independent siRNAs, was sufficient to induce down- and up-regulation of epithelial and mesenchymal markers of EMT, respectively, in the presence of TGF-β (Figure 5D). It is also important to note that transfection of a smaller amount of *PDE1C* siRNAs (20 ng) into TGF-β-treated OSCC cells was sufficient to induce molecular changes to a degree comparable to those with stable expression of miR-133, implying synergistic effects between miR-133/*PDE1C*-siRNA and TGF-β on EMT (Supplementary Figure S10). To further confirm the relevance of PDE1C in development of EMT phenotype in the presence of TGF-β, we adopted a pharmacological alternative of *PDE1C*-siRNA using a non-selective cAMP phosphodiesterase inhibitor, 3-Isobutl-1-methylxanthine (IBMX, 1 mM). Indeed, an administration of IBMX to TGF-β-treated OSCC cells revealed profiles of EMT markers similar to those induced by transfection of *PDE1C*-siRNA (Supplementary Figure S11). Taken together, these data collectively imply a potential regulation of EMT signaling via the activity of a functional network consisting of miR-133 and its target, *PDE1C*.

### Lower expression of miR-133 is correlated with improved survival of human oral cancer patients

**Figure 6:**
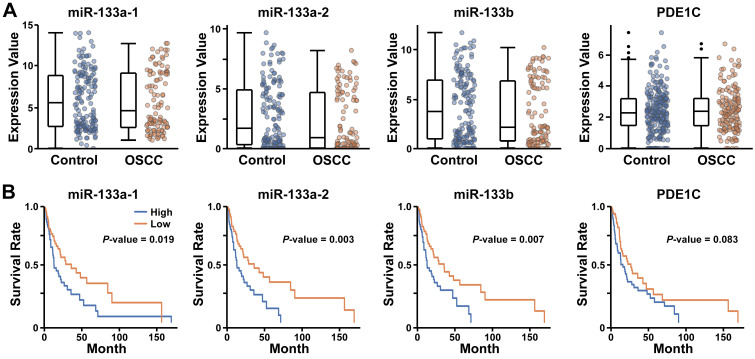
** Enhanced survival of oral cancer patients with lower levels of miR-133 and PDE1C.** (A) The relative expression levels of three different miR-133s (miR-133a-1 (miR-133a-5p), miR-133a-2 (miR-133a-3p) and miR-133b) and *PDE1C* are compared between 236 control and 145 OSCC tumor samples deduced from Broad GDAC Firehose (https://gdac.broadinstitute.org/) (see Materials and Methods for details). (B) The survival rates of OSCC patients with differential expression of miR-133s and *PDE1C* are visualized for comparisons. The significance level of a single analysis is indicated in each plot.

Our findings demonstrating miR-133-dependent facilitation of EMT in human cancer cells raised a possibility that the expression level of miR-133 may be correlated with the prognosis of oral cancer patients. To test this possibility, we analyzed the RNAseq datasets of OSCC selected from Broad GDAC Firehose (https://gdac.broadinstitute.org/) (see Materials and Methods for details). When 236 control and 145 OSCC tumor samples deduced from the collection were analyzed for the expression of miR-133s (miR-133a-1 (miR-133a-5p), miR-133a-2 (miR-133a-3p) and miR-133b), we found a wide range of their expression in both control and OSCC samples, with comparable expression profiles (Figure 6A; *P*-value, 0.578, 0.212 and 0.289 for hsa-miR-133a-1, hsa-miR-133a-2 and hsa-miR-133b, respectively). However, the subsequent survival analyses revealed consistently improved long-term survival of patients with lower expression of miR-133s (Figure 6B). These clinical data are in line with our findings that suggest a potential role of miR-133 in promoting acquisition of aggressive behaviors of oral cancer cells.

In case of *PDE1C*, we found no significant difference in its expression between controls and OSCC patients (Figure 6A; *P*-value, 0.561). While lower expression of *PDE1C* appeared to favor the survival of some OSCC patients at a later stage, it failed to reach a statistical significance in our hands (Figure 6B; *P*-value, 0.083). In addition, the level of *PDE1C* was not clearly associated with the survival rate of overall patients at earlier stages following diagnosis (Figure 6B), raising a possibility of miR-133-dependent regulation of unidentified targets other than *PDE1C* that is likely associated with the prognosis of OSCC patients.

## Discussion

Since its first characterization in early 1900s, *Drosophila melanogaster* has been serving as a versatile alternative animal model that allowed us to investigate vital functions conserved across phyla *in vivo*. With the help of sophisticated genetic tools, it continues to expand its horizon in deciphering molecular networks, of which abnormality may underlie a variety of human disease states. In this study, we adopted a hybrid platform that combined *Drosophila in vivo* and human oral cancer cell-based *in vitro* systems to investigate functional relationships between a specific miRNA and its target relevant to oral cancer. Validation of miR-133-dependent targeting of *PDE1C* in both *Drosophila* wing discs and human oral cancer cells provides a further support to our combinational approach in the process of functional characterization of novel molecules implicated in human diseases. Our results indicate that miR-133-dependent downregulation of *PDE1C* is likely correlated with promoted tissue growth in *Drosophila* as well as accelerated expression of EMT phenotypes in oral cancer cells, presumably underlying more pronounced aggressive cellular behaviors.

Previous studies on miR-133 have been mostly centered around its role as a tumor suppressor in subsets of human malignancies, including gastric cancer 17, glioma 18 and non-small cell lung cancer 19. Furthermore, expression of miR-133 has been frequently linked to reduced cell proliferation and diminished invasive ability of cancer cells via downregulation of *EGFR*, *FOXC1*, *FOXQ1* and *PSEN1* 14, 22-24. In line with these data, miR-133-dependent upregulation of E-cadherin as a readout of attenuated EMT phenotypes has also been documented in gastric and lung cancer cell lines 23, 24. Meanwhile, recent studies on esophageal squamous cell carcinoma and OSCC have suggested miR-133-dependent targeting of *COL1A1* to control invasive and migratory behaviors of cancer cells, along with significantly reduced cell proliferation and enhanced apoptotic activity induced by miR-133 mimics 25, 26. These findings were also coincided with reduced levels of endogenous miR-133 in 20 esophageal and 33 OSCC tumor samples, as well as with a tendency of better prognosis in patients with higher expression of miR-133 25, 26. In contrast, our results clearly point out a possibility of miR-133 as an oncomiR, as evidenced by promoted tissue growth in *Drosophila* wing discs (Figure 1) and by facilitated aggressive cellular behavior in oral cancer cells (Figures 3). Consistently, overexpression of miR-133, presumably via downregulation of *PDE1C*, was able to intensify the molecular signature of EMT (Figures 3-5). For instance, we were able to detect downregulation of E-cadherin in OSCC cells with stable expression of miR-133 (Figures 4 and 5; Supplementary Figures S8 and S10), in contrast to the previous findings in gastric and lung cancer cell lines 23, 24. It should also be noted that, unlike the aforementioned analyses on esophageal and OSCC samples 25, 26, our bioinformatical analysis of the OSCC RNAseq datasets from Broad GDAC Firehose failed to reveal significant differences in the level of miR-133s (Figure 6A; 236 control and 145 tumor samples). However, it clearly projected a poor survival rate in oral cancer patients with higher expression of miR-133 (Figure 6), thus further in line with the more prominent EMT signatures and acquisition of aggressive behaviors induced by miR-133 in oral cancer cells (Figures 3-5).

While the nature of such discrepancies remains unclear and need to be investigated in detail in future studies, some of them may be attributed to different experimental paradigms employed in each study. For instance, most experimental manipulations of miR-133 in other types of cancer cells, including gastric, lung and esophageal cancer cell lines 23, 24, 26, have relied upon transient transfection of miR-133 mimics, while we sought to establish stable OSCC cell lines with enhanced expression of miR-133. As a result, the level of miR-133 upon induced expression could significantly differ among studies. For instance, Chen et al. 24 and Yin et al. 26 have reported around 3- to 4-fold increases in the level of miR-133 following transfection in gastric and esophageal cancer cell lines, respectively. In contrast, we found nearly a 30-fold increase in OSCC cell lines with stable expression of miR-133 (Supplementary Figure S3). Such difference in the level of miR-133 in each system could influence the activity of miR-133-dependent molecular networks involved in the process of EMT and other relevant cancer biology. It should also be noted that we did not observe any changes in the rate of cell proliferation in OSCC cells with stable expression of miR-133 even in the presence of an EMT-promoting signal such as TGF-β (Supplementary Figure S4). This is inconsistent with attenuated cell proliferation and promoted cell death in esophageal and OSCC cells with transient transfection of miR-133 mimics 25, 26, raising a possibility of distinct cellular states examined in these studies. Furthermore, previous studies have identified *FOXQ1*, *PSEN1* and *COL1A1* as the major molecular targets of miR-133 underlying its inhibitory role in EMT and cancer progression 23-26. Here, we propose a novel target of miR-133, *PDE1C*, at the center of mediating EMT phenotypes, which has been rarely recognized as a critical component in this process. Assuming that the final cellular readout in response to miR-133 would be determined by the collective sum of individual molecular activities each targeted by miR-133, functional consequences of miR-133-dependent regulation may depend upon the level of miR-133 and the choice of its main targets as well as their endogenous activity in a specific cellular context.

As stated above, our results demonstrate a central role of PDE1C in the process of EMT in oral cancer (Figure 5). A previous report on glioblastoma multiforme cells suggested PDE1C as an essential component promoting cell proliferation and invasion 43. In human oral melanoma cell lines, vinpocetine and EHNA, inhibitors of phosphodiesterase 1 (PDE1) and PDE2A, respectively, have been shown to inhibit cell growth 44, 45. Notably, downregulation of *PDE1C* by miR-133 in our hands promoted expression of mesenchymal markers in OSCC cells in exchange of downregulation of epithelial cell-biased molecules (Figure 5). Furthermore, our preliminary finding indicates a more pronounced molecular signature of EMT following an administration of IBMX, a cAMP/cGMP phosphodiesterase inhibitor in OSCC cells (Supplementary Figure S11), consistent with the findings in those transfected with *PDE1C*-siRNA (Figure 5; Supplementary Figure S10). In addition, downregulation of *Pde1c* in *Drosophila* wing discs resulted in a noticeable increase in their size via increased cell proliferation (Supplementary Figures S1 and S2), further supporting the idea of PDE1C as an inhibitory signal for cancer progression.

While our data suggestive of accelerated EMT are inconsistent with the previous findings stated above, it should be noted that the activity of PDE1C has never been thoroughly examined in the context of OSCC, the most common form of oral cancer, leaving the functional consequences of its regulation largely unexplored. With the expression profiles and regulatory mechanisms of adenylyl cyclase and PDE diverse among different types of cells, tight regulation of cAMP levels by these enzymes is critical for a wide array of cellular processes, generating the cell- and stimulus-specific responses 46. It is plausible that the level of endogenous PDE1C could vary to a significant degree among different types of cancer cells. Therefore, a systematic experimental approach will be required in future studies in order to delineate cell-specific, cAMP-dependent regulation of biological responses in different types of cancer cells.

Recent research efforts have been made to discover novel drug candidates targeting PDEs in hopes of developing alternative therapeutic options 47-50. As a result of such effort, a limited number of PDE inhibitors are currently in clinical use, including PDE3, PDE4 and PDE5 inhibitors 47. As stated above, our preliminary data indicate pharmacologically induced facilitation of EMT phenotypes in oral cancer cells by an application of a non-selective cAMP/cGMP PDE inhibitor (Supplementary Figure S11). Recently, PDE1 was shown to be specifically targeted by differentiation-inducing factor-1 (DIF-1), a *Dictyostelium discoideum*-derived antitumor agent 51. It will be important to investigate whether treatments of OSCC cells or *Drosophila* larvae with DIF-1 would lead to similar facilitation of EMT phenotypes or tissue growth observed in our study.

Our findings of miR-133-*PDE1C* network-dependent regulation of EMT phenotypes in the presence of TGF-β raise a possibility of functional crosstalk between miR-133- and TGF-β-dependent signaling pathways in oral cancer. In this study, we have utilized TGF-β as a tool to provide a sensitive background to monitor potential changes in the profile of EMT markers. TGF-β is a well-established regulator of transcriptional repressors involved in the expression of EMT phenotypes in a variety of human malignancies 41. Recent reports have suggested both direct and indirect regulation of TGF-β signaling by miRNAs. For instance, the core and downstream signaling components of TGF-β such as the ligand and receptors as well as SMADs can be regulated by the activity of various miRNAs, including miR-200 52, 53. On the other hand, the activity of downstream effectors of TGF-β signaling such as SMADs could influence the biogenesis of miRNAs such as miR-21 via their effects on Drosha-mediated pri-miRNA processing 54, 55. As a result, these bi-directional interactions constitute functional feedback loops to tightly control the activity of miRNA-TGF-β signaling networks 56, 57. A potential regulation of TGF-β signaling by miR-133 has been rarely investigated, with limited numbers of experimental evidence presented in lung cancer cells 23. Our preliminary results did not indicate consistent alterations in the activity of TGF-β signaling pathways, such as phosphorylation of SMADs, in oral cancer cells with relatively high expression of miR-133 (data not shown). Furthermore, a treatment of TGF-β did not trigger further expression of miR-133 in oral cancer cells (Supplementary Figure S12). Thus, it remains unclear whether the bidirectional miR-133-TGF-β signaling networks function in order to regulate the biology of oral cancer cells. Interestingly, our result indicates TGF-β-dependent downregulation of PDE1C, as evidenced by a significant decrease in the level of *PDE1C* mRNA (Figure 5C), in line with a previous report on human alveolar epithelial cells demonstrating TGF-β-dependent downregulation of PDEs, including PDE1A and PDE3A 58. Our subsequent analyses revealed a further decrease in *PDE1C* mRNA upon stable overexpression of miR-133 (Figure 5C), thus suggesting a potential convergence of miR-133- and TGF-β-dependent signaling upon PDE1C during the process of EMT. The role of such convergence on PDE1C-mediated regulation of EMT in the pathophysiology of oral cancer awaits further investigations.

In summary, we demonstrate miR-133-dependent targeting of *PDE1C* in both *Drosophila melanogaster* and human oral cancer cells. Downregulation of *PDE1C* by miR-133 is positively correlated with accelerated expression of EMT, a process central to acquisition of aggressive behaviors of cancer cells. Future analysis on the activity regulation of PDE1C in *Drosophila* and human oral cancer cell platforms will provide a further insight into the molecular mechanisms underlying the pathophysiology of oral cancer.

## Supplementary Material

Supplementary figures.

## Abbreviations

OSCC: oral squamous cell carcinoma
PDEs: Phosphodiesterases
PDE1C: phosphodiesterase type IC
miR-133: microRNA-133
EMT: epithelial-mesenchymal transition
LPS: Lipopolysaccharides
TGF-β: Transforming growth factor- β
Gli3: Glioma-associated Oncogene Family Zinc Finger 3
Runx2: RUNX family transcription factor 2
BMP: Bone morphogenetic protein
EGFR: Epidermal growth factor receptor
FGF1: Fibroblast growth factor 1
IGF1R: Insulin-like growth factor 1 receptor
FOXC1: Forkhead box C1
FOXQ1: Forkhead box Q1
PSEN1: Presenilin 1
EHNA: Erythron-0-(2-hydroxy-3-nonyl) adenine
DIF-1: Differentiation-inducing factor-1
