# Supplementary material for: MicroRNA-133 Targets Phosphodiesterase 1C in Drosophila and Human Oral Cancer Cells to Regulate Epithelial-Mesenchymal Transition
Source: J Cancer. 2021 Jul 3;12(17):5296–309. doi: 10.7150/jca.56138 (PMC8317528; doi:10.7150/jca.56138)
Supplement: Supplementary file 1 — Supplementary figures. [file jcav12p5296s1.pdf]

MicroRNA-133 targets *phosphodiesterase 1C* in *Drosophila* and human oral cancer cells to regulate epithelial-mesenchymal transition

Ji Eun Jung[1,2], Joo Young Lee[3], Hae Ryoun Park[1,2,3,4], Ji Wan Kang[5], Yun Hak Kim[6] and Ji Hye Lee[1,2,3,4*]

*Correspondence: jihyelee@pusan.ac.kr; Tel.: +82-51-510-8259

Supplementary Materials

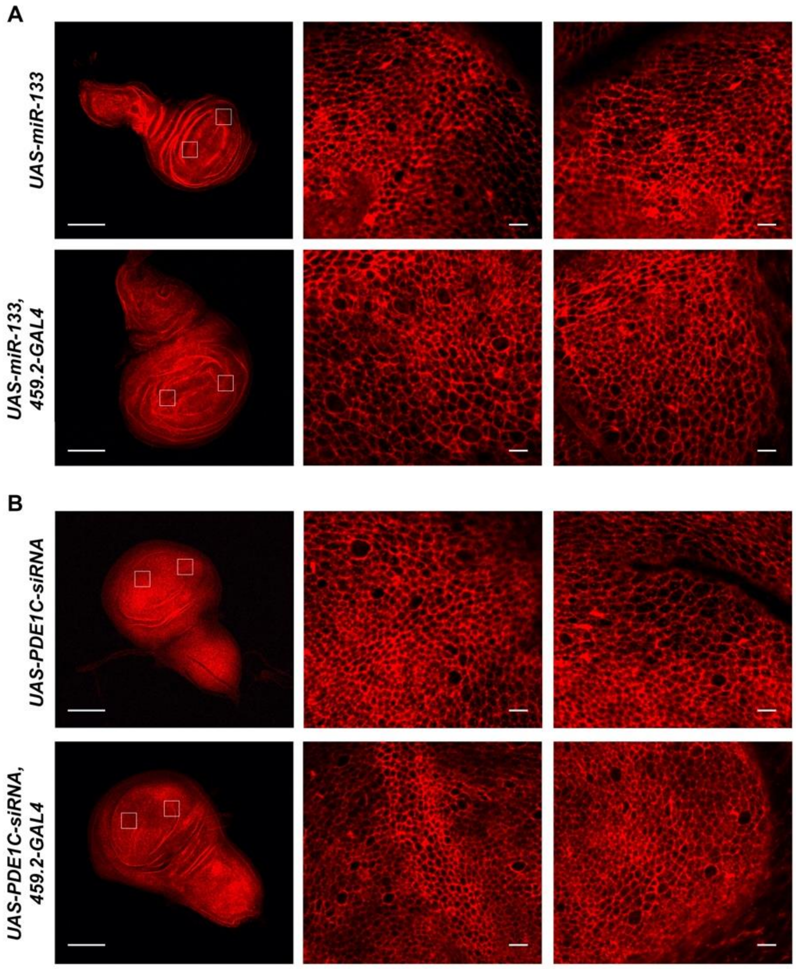

Supplementary Figure S1. Comparison of the size of imaginal wing discs and cells constituting the discs.

Representative images of confocal microscopy are shown to indicate the size of imaginal wing discs and subsets of cells constituting the discs in each genotype indicated. The boundary of cells is visualized with the fluorescent signal of phalloidin. The left panel images were taken at the magnification of 100. The middle and right panels depict the regions indicated with boxes on the left, for which images were taken at the magnification of 1000. Scale bar, 100 and 5 μm for the left and middle/right panels, respectively.

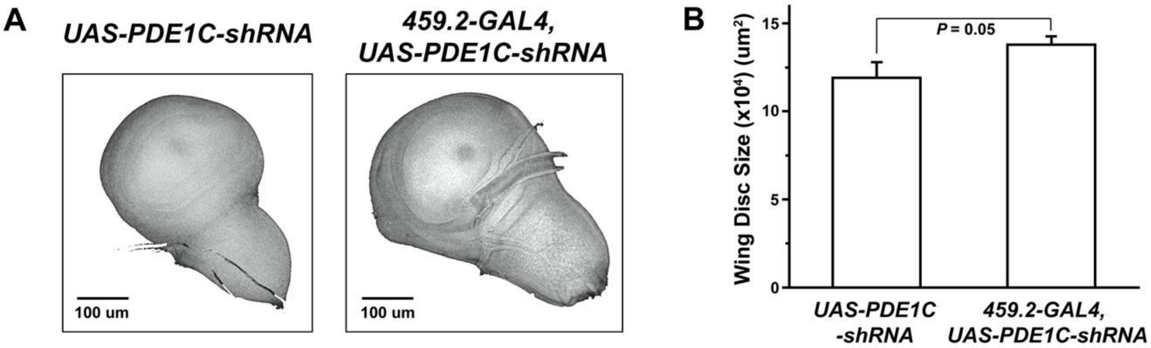

Supplementary Figure S2. Comparison of the size of imaginal wing discs following overexpression of *Drosophila* shRNA against *PDE1C*.

(A) Representative images of *Drosophila* wing discs are shown for those with (right) and without overexpression of shRNA against *PDE1C* (left). Scale bar, 100 μm. (C) The pooled data are shown for measurements of the size of wing discs in each genotype indicated. The number of wing discs examined: 10 and 6 for *UAS-PDE1C-siRNA* with and without *459.2-GAL4*, respectively. Mean ± SEM values are indicated.

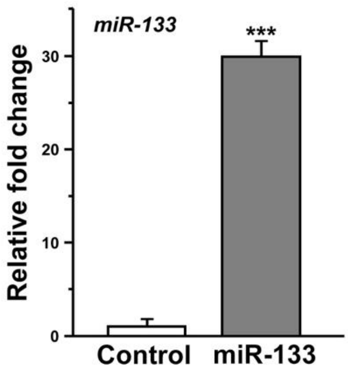

Supplementary Figure S3. Measurement of relative expression levels of miR-133 in SAS cells following stable expression of miR-133.

The pooled data from 11 samples are summarized for the level of miR-133 in SAS OSCC cells with (gray) and without stable expression of miR-133 (white). Mean ± SEM values are indicated. ***, *P*<0.001 for control vs. miR-133.

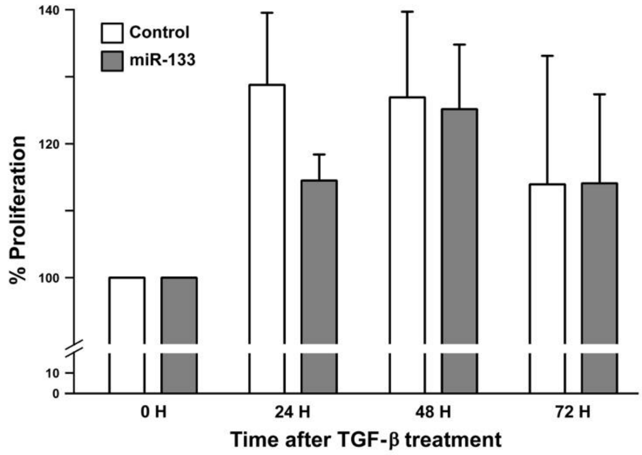

Supplementary Figure S4. Analysis of cell proliferation following stable expression of miR-133 in SAS OSCC cells.

The pooled data from 4 to 8 independent experiments are summarized for the rate of cell proliferation in SAS OSCC cells with (gray) and without stable expression of miR-133 (white) for 72 hours. Mean ± SEM values are indicated.

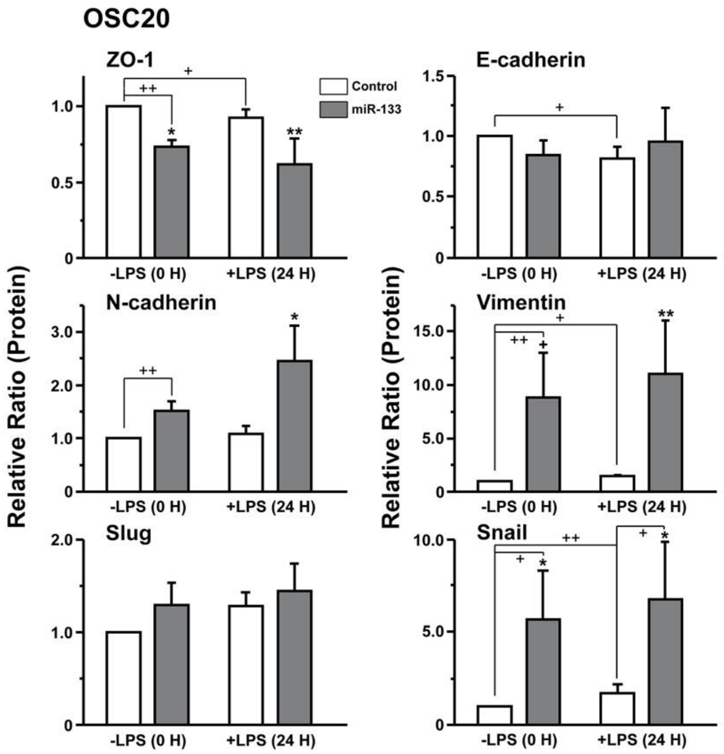

Supplementary Figure S5. Quantitative analysis on the levels of EMT markers in LPS-treated OSC20 cells with stable expression of miR-133.

The pooled data from 4 to 7 western blot analyses are summarized for the relative ratio of protein in each group indicated. For comparison, the ratios were normalized to the value obtained from the control group before a treatment of LPS for each protein probe within the same blot. Mean ± SEM values are indicated. The statistical analyses used area as follows; 1) The Kruskal-Wallis ANOVA is performed, followed by Dunn's test: asterisks (*) for the SAS-control before a LPS treatment vs. other groups, *, P<0.05 and **, P<0.01; 2) Mann-Whitney or two-sample *t*-test is performed ($^{+}$), $^{+}$, P<0.05 and $^{++}$, P<0.01 for SAS-control vs. SAS-miR-133 groups under each experimental condition (-LPS and +LPS).

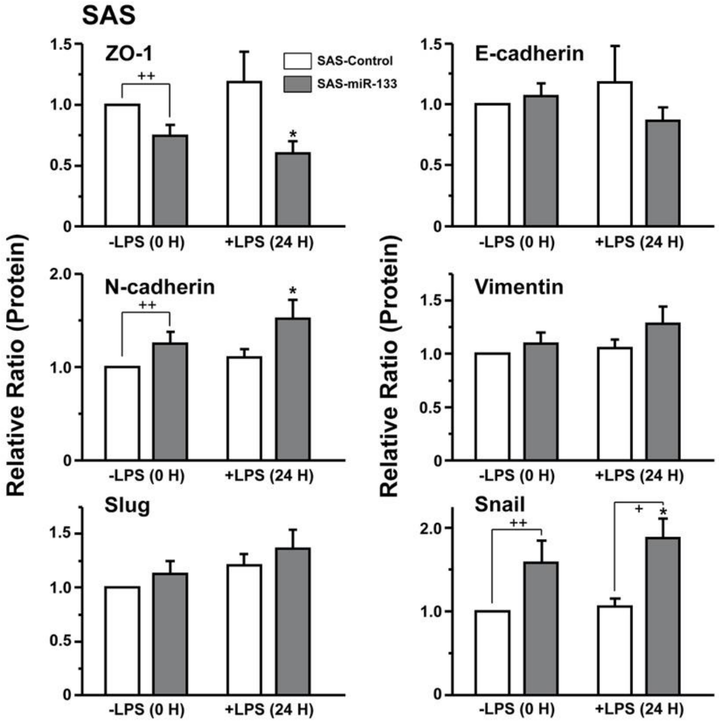

Supplementary Figure S6. Quantitative analysis on the levels of EMT markers in LPS-treated SAS cells with stable expression of miR-133.

The pooled data from 4 to 7 western blot analyses are summarized for the relative ratio of protein in each group indicated. For comparison, the ratios were normalized to the value obtained from the control group before a treatment of LPS for each protein probe within the same blot. Mean ± SEM values are indicated. The statistical analyses used area as follows; 1) The Kruskal-Wallis ANOVA is performed, followed by Dunn's test: asterisks (*) for the SAS-control before a LPS treatment vs. other groups, *, $P<0.05$; 2) Mann-Whitney or two-sample *t*-test is performed ($^{+}$), $^{+}$, $P<0.05$ and $^{++}$, $P<0.01$ for SAS-control vs. SAS-miR-133 groups under each experimental condition (-LPS and +LPS).

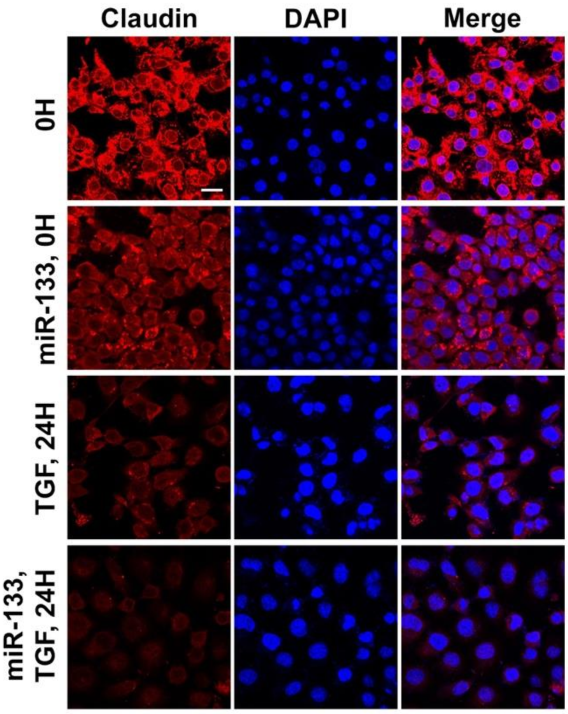

Supplementary Figure S7. Immunofluorescent analysis of an epithelial marker, claudin, in TGF-β-treated SAS cells with stable expression of miR-133.

Representative images of confocal microscopy are shown to indicate the level and localization of immunoreactivity signals against claudin, an epithelial marker critical for maintaining the cell-cell junctions, in control and miR-133-expressing SAS cells before and after a treatment of TGF-β for 24 hours. Scale bar, 20 μm, for all images.

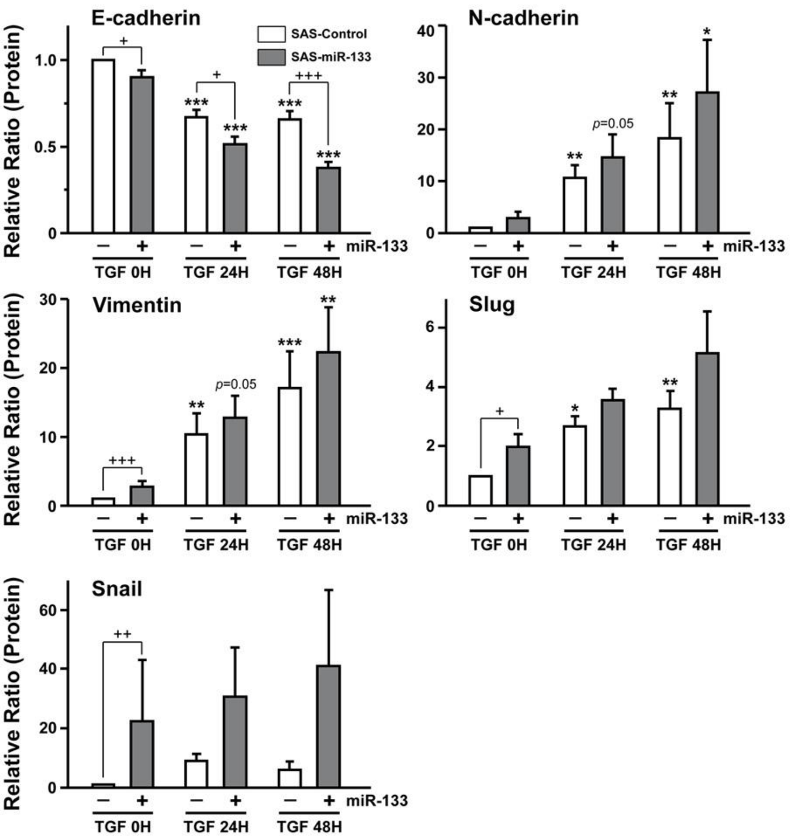

Supplementary Figure S8. Quantitative analysis on the levels of EMT markers in TGF-β-treated SAS cells with stable expression of miR-133.

The pooled data from 5 to 13 western blot analyses are summarized for the relative ratio of protein in each group indicated. For comparison, the ratios were normalized to the value obtained from the control group before a treatment of TGF-β for each protein probe within the same blot. The statistical analyses used are as follows; 1) The Kruskal-Wallis ANOVA is performed, followed by Dunn's test: asterisks (*) for each group (SAS-control and SAS-miR-133) at TGF 0H vs. other time points, *, P<0.05, **, P<0.01 and ***, P<0.001; 2) Mann-Whitney or two-sample *t*-test is performed ($^{+}$), $^{+}$, P<0.05, $^{++}$, P<0.01 and $^{+++}$, P<0.001 for SAS-control vs. SAS-miR-133 groups at each time point.

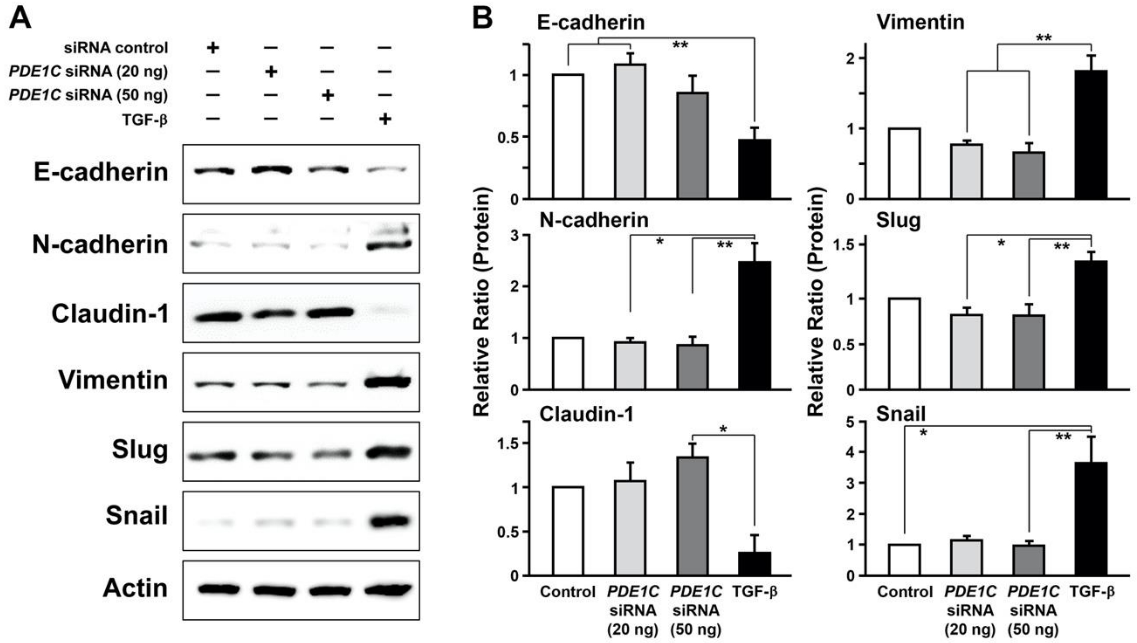

Supplementary Figure S9. The effect of overexpressed *PDE1C* siRNA on the levels of EMT markers in SAS cells.

(A) The levels of molecular markers characteristic of EMT are indicated by a representative western blot analysis for SAS cells in the presence and absence of siRNA against *PDE1C*. The results from the group treated with TGF-β are included as a positive control. (B) The pooled data from 3 to 8 western blot analyses are summarized for the relative ratio of protein in each group indicated. For comparison, the ratios were normalized to the value obtained from the control group before a treatment of siRNA against *PDE1C* or TGF-β for each protein probe within the same blot. The Kruskal-Wallis ANOVA is performed, followed by Dunn's test. Mean ± SEM values are indicated. *, P<0.05 and **, P<0.01 for the experimental conditions indicated.

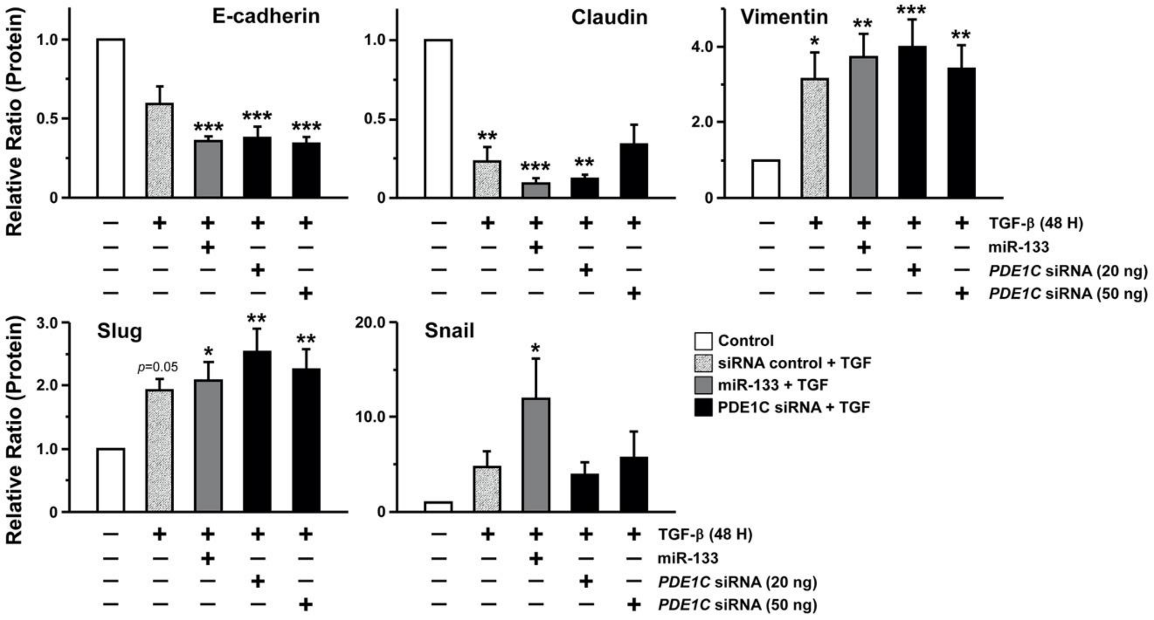

Supplementary Figure S10. Quantitative analysis on the levels of EMT markers in TGF-β-treated SAS cells with stable expression of miR-133 or transfection of siRNAs targeting *PDE1C*.

The pooled data from 6 to 10 western blot analyses are summarized for the relative ratio of protein in each group indicated. For comparison, the ratios were normalized to the value obtained from the control group before a treatment of TGF-β for each protein probe within the same blot. The Kruskal-Wallis ANOVA is performed, followed by Dunn's test. Mean ± SEM values are indicated. *, $P<0.05$, **, $P<0.01$ and ***, $P<0.001$, control vs. the experimental condition indicated.

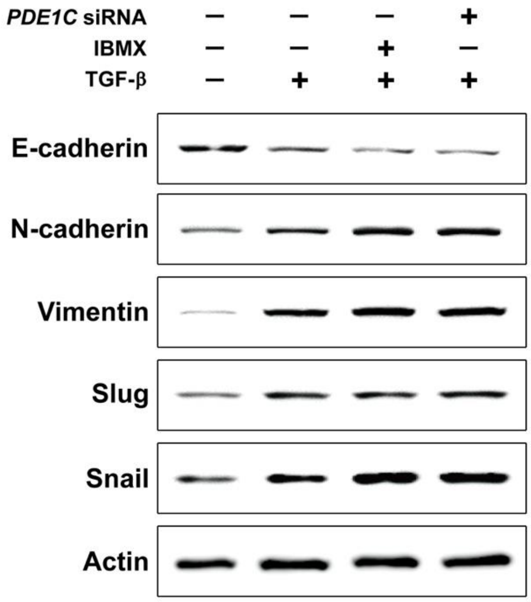

Supplementary Figure S11. The effect of IBMX on the levels of EMT markers in SAS cells treated with TGF-β.

A representative western blot result is shown for expression of EMT markers in SAS OSCC cells treated with IBMX (3-Isobutyl-1-methylxanthine, 1 mM), a non-selective inhibitor of cAMP phosphodiesterase, or *PDE1C* siRNA (50 μg) in the absence or presence of TGF-β for 48 hours.

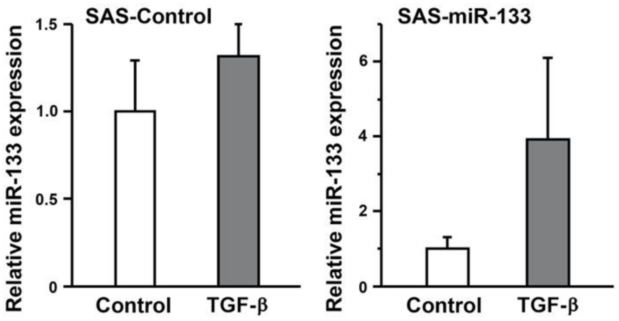

Supplementary Figure S12. Measurement of relative expression levels of miR-133 in SAS cells following an application of TGF-β.

The levels of miR-133 are compared between SAS OSCC cells with (right) and without stable expression of miR-133 (left) before (white) and after a treatment of TGF-β (gray). Mean ± SEM values are indicated.
